# Revisiting the Measurement of Anomie

**DOI:** 10.1371/journal.pone.0158370

**Published:** 2016-07-06

**Authors:** Ali Teymoori, Jolanda Jetten, Brock Bastian, Amarina Ariyanto, Frédérique Autin, Nadia Ayub, Constantina Badea, Tomasz Besta, Fabrizio Butera, Rui Costa-Lopes, Lijuan Cui, Carole Fantini, Gillian Finchilescu, Lowell Gaertner, Mario Gollwitzer, Ángel Gómez, Roberto González, Ying Yi Hong, Dorthe Høj Jensen, Minoru Karasawa, Thomas Kessler, Olivier Klein, Marcus Lima, Tuuli Anna Mähönen, Laura Megevand, Thomas Morton, Paola Paladino, Tibor Polya, Aleksejs Ruza, Wan Shahrazad, Sushama Sharma, Ana Raquel Torres, Anne Marthe van der Bles, Michael Wohl

**Affiliations:** 1 University of Queensland, Brisbane, Australia; 2 University of Melbourne, Melbourne, Australia; 3 University of Indonesia, Jakarta, Indonesia; 4 University of Lausanne, Lausanne, Switzerland; 5 Institute of Business Management, Karachi, Pakistan; 6 Université Paris Ouest Nanterre La Défense, Paris, France; 7 University of Gdańsk, Gdańsk, Poland; 8 University of Lisbon, Lisbon, Portugal; 9 East China Normal University, Shanghai, China; 10 Université Libre de Bruxelles, Brussels, Belgium; 11 University of the Witwatersrand, Johannesburg, South Africa; 12 University of Tennessee, Knoxville, Tennessee, United States of America; 13 Philipps University Marburg, Marburg, Germany; 14 Universidad Nacional de Educación a Distancia, UNED, Madrid, Spain; 15 Pontificia Universidad Católica de Chile, Santiago, Chile; 16 Nanyang Technological University, Singapore, Singapore; 17 Aarhus University, Aarhus, Denmark; 18 Nagoya University, Nagoya, Japan; 19 University of Jena, Jena, Thuringia, Germany; 20 Federal University of Sergipe, Sergipe, Brazil; 21 University of Helsinki, Helsinki, Finland; 22 ISCTE—University Institute of Lisbon, Lisbon, Portugal; 23 University of Exeter, Exeter, England, United Kingdom; 24 University of Trento, Trento, Italy; 25 Hungarian Academy of Sciences, Budapest, Hungary; 26 Daugavpils University, Daugavpils, Latvia; 27 National University of Malaysia, Bangi, Malaysia; 28 Kurukshetra University, Kurukshetra, Haryana, India; 29 Federal University of Paraíba, João Pessoa, Brazil; 30 University of Groningen, Groningen, Netherlands; 31 Carleton University, Ottawa, Ontario, Canada; Mälardalen University, SWEDEN

## Abstract

Sociologists coined the term “anomie” to describe societies that are characterized by disintegration and deregulation. Extending beyond conceptualizations of anomie that conflate the measurements of anomie as ‘a state of society’ *and* as a ‘state of mind’, we disentangle these conceptualizations and develop an analysis and measure of this phenomenon focusing on anomie as a perception of the ‘state of society’. We propose that anomie encompasses two dimensions: a perceived breakdown in social fabric (i.e., disintegration as lack of trust and erosion of moral standards) and a perceived breakdown in leadership (i.e., deregulation as lack of legitimacy and effectiveness of leadership). Across six studies we present evidence for the validity of the new measure, the Perception of Anomie Scale (PAS). Studies 1a and 1b provide evidence for the proposed factor structure and internal consistency of PAS. Studies 2a-c provide evidence of convergent and discriminant validity. Finally, assessing PAS in 28 countries, we show that PAS correlates with national indicators of societal functioning and that PAS predicts national identification and well-being (Studies 3a & 3b). The broader implications of the anomie construct for the study of group processes are discussed.

## Introduction

For more than a decade after the fall of the Soviet Union, many Eastern European countries struggled with social and political instability. Consider Bulgaria, a country that changed its entire political system in 1989. The term anomie was used to describe the state of society in Bulgaria that emerged after the economy had collapsed and the political system struggled to respond [1, 2]. Anomie has also been used to describe the state of society in countries undergoing massive structural change (e.g., Iran, see [3, 4]), countries that face major social or economic crises [5], countries with a long history of war (e.g., Yugoslavia, Iraq, Afghanistan, see [6, 7]), or societies that face civil unrest (e.g., South Africa, [8]). Anomie has even been used to describe social contexts with relative prosperity, but where income inequality has eroded social capital and trust (e.g., the U.S., [9, 10, 11]), or where rapid economic growth has created instability and unrest (e.g., China, [12]).

Despite the fact that anomie is a common experience that many people and societies in the world today share, to date there is no uniform conceptualization and operationalization of this construct. In this paper, we first review the previous conceptualizations and operationalizations of anomie and highlight their shortcomings. Starting from an operationalization of anomie as a perception of the state of society, we develop a scale that disentangles anomie as a state of society from its outcomes at the individual level. In this way, we equip the field with a tool that can be used to develop better insights into the nature of anomie and the experience of those who live in a state of anomie.

### Anomie: The Concept and its Measurement

Even though anomie has been conceptualized in different ways, perhaps the most well-known approach is to define anomie as a state of society [9–11, 13]. Durkheim [13] proposed that anomie involves the breakdown of social regulation and the rise of moral disruption. Merton [9, 10] extended this thinking and proposed that anomie emerges from the discrepancy between the cultural aspirations of people within a society and the legitimate means available to those people to achieve them. Still focusing on the state of society, Messner and Rosenfeld [11] took a slightly different approach to Merton and focused instead on anomie as a cultural obsession with economic success, manifesting itself as a set of cultural values associated with individualism, achievement orientation, and fetishism of money.

Departing somewhat from this well-known work, some have defined anomie as a state of mind. Scholars in this tradition have focused on anomie as an individual’s sense of self-to-other alienation or distance [14], or as a set of beliefs, feelings and attitudes in the individual’s mind [15, 16]. In general, these approaches to anomie revolve around a psychological state that can be characterized as a tendency to be self-interested [17], to reject social norms [18, 19], or to feel estranged or isolated from society [14, 20, 21]. In this conceptualization, anomie may also include a sense that life is meaningless [21, 22], where feelings of purposelessness or powerlessness dominate (for reviews, see [19, 23]).

These different conceptualizations have informed different operationalizations of the concept of anomie, each with their own specific shortcomings. Relying on a conceptualization of anomie as a state of mind has led to the problem that the measurement of anomie is identical to the measurement of its individual-level outcomes (for a similar argument, see [19, 24, 25]). For instance, anomie is inferred from individuals’ self-reported loneliness (e.g., “I feel all alone these days”, see [20, 26, 27]), frustration (e.g., “I often feel awkward and out of place”, see [16, 28]), or powerlessness (e.g., “I have no control over my destiny”, see [27]). In general, the conceptualization of anomie as a state of mind has yielded measures that depict personal despair and confusion, frustration, meaninglessness, isolation, and powerlessness [19, 23, 24, 29]. Arguably, by focusing on these individual-level symptoms or outcomes of anomie, the analysis of the anomie phenomenon itself as a state of society has been overlooked in empirical studies.

Similar measurement issues arose for those who proposed a conceptualization of anomie as a state of society. These researchers typically operationalized and measured anomie by focusing on the hypothesized outcomes (perhaps due to the difficulty of measuring the social structural context directly, see [30]). For instance, researchers have measured anomie as the suicide or homicide rate within a particular community or society [11, 31], the endorsement of cultural values such as individualism and fetishism of money [32], or as uncertainty (e.g., “You can never be certain of anything in life”, [19]). Therefore, both conceptualisations have generated a mismatch between the conceptual and operational definitions of anomie.

Given that collective-level phenomena cannot be understood solely by studying individual-level processes and outcomes [33–36], we argue that an appropriate measure of anomie must distinguish between the collective-level phenomenon of anomie from its individual-level outcomes.

We argue that anomie should be measured consistently and solely as a perception of society, specifically a perception that the social and political conditions in society are crumbling. It is important to note that in this operationalization we measure anomie not as a personal belief or feeling but rather as a reflection of the societal state in individuals’ minds. It is also important to note that anomie is not about the objective conditions of society but rather the perceived conditions of society. That is, while there may be objective triggers for anomie (rapid societal changes, [1, 2]; rapid economic growth or crises, [5]; economic inequality, [12]; domination of cultural values on far-reaching economic goals/aspirations or high materialism, [9–11]; war or civil conflict, [6–8]), these triggers lead to changes in perception that are then communicated intersubjectively within the given context. That is, the formation of a collective phenomenon depends on the extent to which individuals intersubjectively construct a shared perception of their social world [37–39]. Our approach, therefore, focuses on the perceived state of society and we argue that such perceptions are the basis from which individually-held beliefs shape, and are influenced by, the collective consciousness (e.g., see [13, 40, 41]).

### Anomie as a Perceived State of Society

In line with classic theorizing on anomie [9, 10, 13], in which social integration (e.g., strong social fabric) and social regulation are considered to be two key components of a healthy society, we define anomie as a perception that a particular society has become disintegrated and disregulated [42]. Extending classic theorizing and linking it to recent social and behavioral research, we propose that disintegration involves a perception that society’s social fabric is breaking down, including a perceived lack of trust and of moral standards [43–45]. Disregulation, on the other hand, involves a perception that leadership of a given society is breaking down, that it is illegitimate and ineffective and that leaders no longer follow fair decision-making processes [46–50], including the perception that leaders do not represent and protect all society members nor distribute resources fairly, and are ineffective in facilitating the collective good [47–49].

We argue that the two dimensions of anomie, breakdown in social fabric and breakdown of leadership, are highly interrelated and can be mutually reinforcing; when one dimension breaks down, it will place additional weight on the other. More specifically, when leaders are viewed as incapable of managing problems within society (ineffective leadership, for reviews, see [50, 51, 52, 53]), and as not representative of the society (illegitimate leadership, see [51, 54]), the cohesiveness of the broader social environment will be undermined. That is, many people feel that they are outsiders who do not receive fair and just consideration, a sentiment that undermines a sense of belonging within the community. Therefore, breakdown in leadership can lead to breakdown in social fabric. In a similar vein, Rothstein and Eek [55] found that when trust in authorities diminishes, general trust in others is also eroded (also see [56]). Similarly, when the social fabric is perceived to be breaking down it becomes more difficult to choose (legitimate) leaders who are representative of all [47, 51, 57].

Thus, anomie arises when these two dimensions of a functioning society–effective leadership and strong social fabric–are perceived to be eroding. That is, we argue that anomie emerges when both a breakdown in leadership and a breakdown of social fabric co-occur.

Concerning individual-level outcomes, we argue that anomie primarily undermines well-being and life satisfaction [58–60] and that it reduces happiness [61]. This is because individuals feel helpless and hopeless in their ability to work toward their desired goals [62–64]. Additionally, anomie perceptions should be associated with withdrawal from the broader social context, including dis-identification with the superordinate group, or lower national identification [14, 65, 66]. This is because illegitimate and ineffective leaders weaken trust, leading to a rapid decline in agreed upon moral norms which in turn damages cooperation within groups, reducing engagement and identification [67–71] and creating schisms and fragmentation that further perpetuate the withdrawal of individuals from society at large [72, 73]. For instance, anomie was found to be high in the Netherlands during the mid-80s [74] and in post-communist Bulgaria [1]; according to these researchers, this high anomie was one of the main reasons for political apathy and social withdrawal in these countries.

## The Current Research

Across six studies we developed and validated a new scale of anomie—labeled the Perception of Anomie Scale (PAS)—which operationalizes anomie as a perception of the state of society encompassing two dimensions: the perceived breakdown of leadership and the perceived breakdown of social fabric. In our measure of anomie, participants were asked to think about their country and consider their agreement with a range of statements describing the perceptions of most individuals in their society (for a similar approach, see [27]).

Three series of studies were conducted to develop PAS and examine its psychometric properties. Specifically, an initial pool of items was identified using exploratory factor analysis (Study 1a) after which we confirmed the factor structure of PAS (Study 1b) and examined its convergent and discriminant validity (Studies 2a-2c).

Studies 3a and 3b examined PAS in 28 countries. In both of Studies 3a and 3b, in addition to the analysis of individuals’ responses to PAS, we used multilevel modelling which aggregates the individuals’ ratings at the country level, thereby capturing perceived anomie at the group level. In Study 3a, we assessed whether anomie scores vary predictably by country and determined whether anomie, as measured by PAS, is associated with indicators of a nation’s economic and social condition, standard of living, and level of corruption. Finally, in Study 3b, we focused on the predictive validity of PAS and examined whether anomie predicts well-being and national identification.

## Scale Construction and Psychometric Analysis: Studies 1a-1b

The aim of Studies 1a and 1b was to select scale items that best captured anomie in terms of its two dimensions, breakdown of social fabric and breakdown of leadership. To achieve this, we conducted exploratory factor analysis (Study 1a) and confirmatory factor analysis (Study 1b) using two separate samples.

### Item generation procedure

Based on our theoretical framework, we generated items intended to capture two aspects of a perceived breakdown in social fabric (perceptions of moral decline and lack of trust) and two aspects of perceived breakdown in leadership (lack of legitimacy and effectiveness). Item generation was in part guided by our conceptual model and in part based on items adapted from previous scales. In some cases we modified items to update the wording. For instance, in an attempt to modernize and minimize items’ length and syntactic complexity, Srole’s [14] item “there’s little use writing to public officials because they aren’t really interested in the problems of the average man”, was changed to “politicians don’t care about the problems of the average person”. Out of the 32 items, 16 items were adapted from previous scales [14, 20, 23, 24, 27, 32, 75–79], and a further 16 items were generated by the authors. Item order was randomized to cancel out any sequence effect (7 items for moral decline, 7 items for lack of trust, 11 items for lack of effectiveness of leadership, and 7 items for lack of legitimacy of leadership).

We asked participants to indicate to what extent most others within their society would agree or disagree with each of the statements on a seven-point Likert-type format from 1 (*strongly disagree*) to 7 (s*trongly agree*). Items were keyed both negatively (13 items) and positively (19 items) to minimize response bias. We reverse-scored negatively keyed phrases to create a total score for anomie that can also be broken down into two subscales capturing perceptions of the social fabric and leadership as separate factors. Higher mean scores indicated perceptions of higher anomie.

### Ethics statement

Both studies obtained ethical clearance from the School of Psychology at the University of Queensland. Before completing the questionnaire, participants were informed about the aims of the study. To indicate their consent, participants ticked a box and they were reminded that they were free to withdraw at any stage without penalty. Participants were debriefed at the end of the survey.

### Participants

In Study 1a, participants were 199 first year psychology students from the University of Queensland. They ranged in age from 16 to 48 (*M* = 19.58; *SD* = 4.96) including 149 females, 48 males, and two who did not report their gender. For Study 1b, 214 US citizens were recruited through Amazon’s Mechanical Turk (MTurk, see [80]). Their age ranged from 18 to 80 (*M* = 36.54; *SD* = 11.96) with 106 females and 108 males.

### Results

#### Exploratory factor analysis

Initially, we checked the factorability of the 32 items of anomie in the Australian sample. We found that the items were inter-correlated, with 107 correlations among 32 items that were higher than .30, suggesting reasonable factorability [81]. The Kaiser-Meyer-Olkin (KMO) measure of sampling adequacy was .86 (acceptable coefficient is .60 and higher) and the Bartlett’s test of sphericity was significant (*χ*^*2*^ (666) = 2067.35, *p* < .001). Both of these suggest good factorability of the correlation matrix and adequacy of the number of participants for factor analysis [81, 82].

Our exploratory factor analysis consisted of three steps. First, we conducted an exploratory factor analysis (unrotated Principal Axis Factoring [PAF], see [83]) and dropped two items that failed to load. Second, exploratory factor analysis (direct oblimin rotation, see [81, 82]; Principal Axis Factoring [PAF], see [83]) on the remaining 30 items led us to drop another 15 items that had high cross-loadings (higher than .30 loadings) on different factors. We chose direct oblimin rotation because, theoretically, we would expect the components of anomie to be related.

This analysis revealed two factors (as indicated by inspection of the scree plot) with high eigenvalues (factor 1 = 3.96 and factor 2 = 1.82). The two-factor structure was consistent with our theoretical model [42] and the two factors clearly represented the two theoretical dimensions of breakdown of social fabric and breakdown of leadership. Finally, in an attempt to (a) create an economical and adequate measure, and (b) balance the two components of anomie without prioritizing one over the other, we aimed to extract the same number of items to tap the two components of each dimension of anomie (i.e., trust and moral decline for breakdown of social fabric, and illegitimacy and ineffectiveness for breakdown of leadership). From the remaining 15 items, we therefore deleted the three items with the lowest loadings.

A factor analysis on the remaining 12 items revealed a two-factor structure, explaining 43% of the variance and all items loading above .40. The first factor consisted of items relating to the breakdown in social fabric (lack of trust and moral decline) and the second factor consisted of items relating to the breakdown in leadership (lack of regulation and lack of legitimacy). The items, their factor loadings, and the origin of adapted items are presented in Table 1. Cronbach’s alpha indicated acceptable internal reliability for breakdown in social fabric (6 items, α = .69), and breakdown in leadership (6 items, α = .74, and for the full anomie scale, α = .77). The two dimensions of PAS were significantly but not highly correlated (*r* = .35, *p* < .001).

**Table 1:** The Items with Factor Loadings.

**Items**	Factor Loadings	
**Instruction: Think of Australian society and indicate to what extent do you agree with the following statements?**		
**In Australia today........**		
**Breakdown of social fabric**		
1. People think that there are no clear moral standards to follow. (+) (Moral decline, adapted from [23]).	.68	
2. Everyone thinks of himself/herself and does not help others in need. (+) (Trust, adapted from [77])	.66	
3. Most of people think that if something works, it doesn’t really matter whether it is right or wrong. (+) (Moral decline, adapted from [78])	.65	
4. People do not know who they can trust and rely on. (+) (Trust, adapted from [14, 75, 77])	.62	
5. Most of the people think that honesty doesn’t work all the time; dishonesty is sometimes a better approach to get ahead. (+) (Moral decline, adapted from [78])	.62	
6. People are cooperative. (-) (Trust, adapted from [20])	.48	
**Breakdown of Leadership**		
7. The government works towards the welfare of people. (-) (Effectiveness, adapted from [76])		.75
8. The government is legitimate. (-) (Legitimacy)		.74
9. The government uses its power legitimately (-) (Legitimacy)		.73
10. Politicians don’t care about the problems of average person. (+)(Effectiveness, adapted from [14, 75–77])		.70
11. The government laws and policies are effective (-) (Effectiveness)		.66
12. Some laws are not fair. (+) (Legitimacy, adapted from [76, 78])		.41

#### Confirmatory factor analysis

We next conducted a confirmatory factor analysis (CFA) using Analysis of Moment Structure (AMOS) on the US sample (Study 1b). Although the Chi Square was significant (*χ*^*2*^*/df* = 1.83, *p* < .001), the two-factorial model resulted in acceptable fit indices, mostly exceeding the .93 benchmark (comparative fit index [CFI] = .96; incremental fit index [IFI] = .96; goodness of fit index [GFI] = .94; Tucker-Lewis index [TLI] = .95; normed fit index [NFI] = .92) and the residual index falling below the .08 benchmark (root mean square error of approximation [RMSEA] = .06). All factor loadings were above .60 (for breakdown of social fabric: .61-.77; for breakdown of leadership: .60-.88). The indicators of the model fit and factor loadings of the items confirm the suitability of the two-factorial model for PAS.

To examine whether or not the two-factorial model was better than a one-factorial solution, we conducted another confirmatory factor analysis combining the two dimensions into a one-factorial structure which resulted in poorer factor loadings (from .41 to .81 with 5 items loading between .41 to .50) and poor model fit (*χ*^*2*^*/df* = 6.23; CFI = .74; IFI = .74; GFI = .73; TLI = .68; NFI = .71; RMSEA = .15). The result of chi-square difference test of competing models shows that the two models are significantly different, and the two-factorial model significantly improves the fit (*χ*^*2*^[5] = 246.83, *p* < .001). The Akaike Information Criterion (AIC) for two-factorial structure (AIC = 147.688) is also much lower than the one-factorial structure (AIC = 384.522) confirming that the former is a better model fit.

In Study 1b, the internal reliability of PAS was satisfactory, with Cronbach’s alphas of .81 for breakdown in social fabric, .87 for breakdown in leadership, and .88 for the whole PAS. The two dimensions were also significantly correlated, *r* = .55, *p* < .001.

### Discussion

In Studies 1a and 1b, we developed a new measure of anomie (PAS) using both exploratory and confirmatory factor analysis. Consistent with previous conceptual theorizing (see [13, 84]), PAS captures two important dimensions related to anomie, breakdown in social fabric and breakdown of leadership. We were also able to develop an internally consistent measure to capture individual perceptions of anomie as a state of society. We next turn to an examination of the validity of the newly developed scale, assessing the convergent validity (Studies 2a-2b) and discriminant validity (Study 2c) of PAS.

## Convergent Validity: Studies 2a-2b

In the next two studies, as an assessment of convergent validity, we examined the relationships between PAS and distinct but theoretically related measures. Anomie has been associated with a sense of collective helplessness [21, 22] and collective hopelessness [1, 63]. Some have argued that anomie reduces confidence in society, lowering perceived cohesion [13, 85, 86], and increasing a belief that the world is dangerous and threatening [25, 85]. These constructs are key features of an anomic society [9, 10] and should therefore correlate with PAS.

In Study 2a, we focused on convergent validity in an online sample from the US examining the relationship between PAS and related constructs including collective helplessness and hopelessness, social cohesion, and dangerous and threatening worldview. Extending on Study 2a, in Study 2b we aimed to replicate this same pattern of relationships in an Australian community sample.

### Ethics statement

Both studies obtained ethical clearance from the School of Psychology at the University of Queensland. The studies’ procedures and aims were explained in an information sheet and participants were asked to tick a box to indicate their consent to participate. Participants were informed that participation was voluntary and that they were free to withdraw from the study at any time without penalty. They were debriefed at the end of the survey.

### Participants

In Study 2a, we recruited 149 participants through Mechanical Turk. Participants were US citizens and their ages ranged from 18 to 66 (*Mean* = 35.07; *SD* = 11.69; 73 females). For Study 2b, 617 Australian participants were recruited using the Taverner social research institution platform (an independent Australian social research company). Data was collected online from a pre-recruited panel of people across Australia. Participants were selected by invitation, with the aim to achieve a representative sample according to age, gender and location (metropolitan and non-metropolitan per state). Participants included 325 females and 292 males and their ages ranged from 18 to 76 (*Mean* = 40.93, *SD* = 12.81).

### Measures

*Collective hopelessness and helplessness* was measured with six items, three items to tap each dimension. We developed the items and instructed participants to imagine their society (i.e., North America, Australia) while responding to items that tapped feelings of collective helplessness and hopelessness. Collective helplessness consisted of three items (“At the moment, people in America/Australia feel helpless”, “Americans/Australians feel that there is not much they can do to change many important things in society”, and “Americans/Australians feel helpless in dealing with the problems around them”). Collective hopelessness also consisted of three items (“Americans/Australians look forward to the future without hope”, “At the moment, people in America/Australia feel hopeless”, and “Americans/Australians have great faith in the future”, reversed scored). Participants responded on a 7-point scale (1 = *strongly disagree;* 7 = *strongly agree*). The scale had good internal consistency (α = .83 and .88 for the North American and Australian sample, respectively), with higher scores indicating higher feelings of collective helplessness and hopelessness.

*Social cohesion* was only measured in the North American sample (Study 2a) using five items tapping community cohesion and trust (e.g., “people are willing to help their neighbors” and “people here generally don’t get along with each other” [reversed], [87, 88]). Participants responded using a 5-point scale (1 = *strongly disagree*; 5 = *strongly agree*). Higher scores indicated higher perceived cohesion within society (α = .69).

*Dangerous and threatening worldview* was measured using a short version of the dangerous and threatening worldview scale that included six items (e.g., “there are many dangerous people in our society who will attack someone out of pure meanness”, [89, 90]). Participants responded using a 7-point scale (1 = *strongly disagree*; 7 = *strongly agree*). The instructions asked participants to what extent they thought Americans/Australians agreed with each statement. Higher scores represented an increased perception that the society was dangerous and threatening (α = .81, .75 for the North American and Australian sample, respectively).

### Results

Table 2 shows the descriptive statistics and also the relationships between PAS and other constructs. In Study 2a, as expected, PAS showed significant (moderate to high in strength) positive relationships with collective helplessness and hopelessness (*r* = .58, *p* < .001), and perceived dangerous and threatening worldview (*r* = .48, *p* < .001). A significant negative relationship (of moderate strength) was found with social cohesion (*r* = -.45, *p* < .001). In Study 2b, we replicated the results of Study 2a, obtaining significant and high positive correlations between PAS and collective helplessness and hopelessness (*r* = .63, *p* < .001) and dangerous and threatening worldview (*r* = .54, *p* < .001).

**Table 2:** Descriptives and bivariate correlations.

	**US sample (Study 2a), N = 149**					
	**Descriptive**			**Correlation**		
	**Mean**	**SD**	**α**	**BSF**	**BL**	**Anomie**
Breakdown of social fabric	4.02	.92	.74	-	-	-
Breakdown of leadership	4.95	1.06	.84	.31***	-	-
Anomie	4.48	.80	.81	.78***	.84***	-
Collective Helplessness and hopelessness	4.55	1.03	.83	.34***	.58***	.58***
Social cohesion	3.28	.58	.69	-.57***	-.18*	-.45***
Dangerous Worldview	4.25	1.09	.81	.44***	.35***	.48***
	**Australian community sample (Study 2b), N = 617**					
	**Descriptive**			**Correlation**		
	**Mean**	**SD**	**Mean**	**BSF**	**BL**	**Anomie**
Breakdown of social fabric	4.24	1.06	.83	-	-	-
Breakdown of leadership	4.49	1.16	.84	.31***	-	-
Anomie	4.36	.90	.84	.79***	.83***	-
Collective Helplessness and hopelessness	4.31	1.15	.88	.56***	.47***	.63***
Dangerous Worldview	4.21	1.02	.75	.49***	.39***	.54***

BSF, breakdown of social fabric; BL, breakdown of leadership.

* at *p* < .05

*** at *p* < .001.

### Discussion

Providing evidence for the convergent validity of PAS in two samples from North America and Australia, we found that PAS was positively related to collective helplessness and hopelessness and positively related to perceptions that the world is a dangerous and threatening place. Furthermore, in the North American sample we found that PAS was associated with lower levels of social cohesion. These findings confirm the convergent validity of our scale.

## Convergent and Discriminant Validity: Study 2c

In Study 2c, we extended our analysis of convergent validity by comparing PAS to previous measures of anomie. As noted in the introduction, existing measures of anomie focus mainly on assessing the individual-level outcomes of anomie (see [11, 19, 23, 24, 25, 29, 31, 32]). Given that we operationalize PAS as the perception of the state of society, our measure should be distinct from but also related to previous anomie scales such as those used by Srole [14] and Agnew [75], both of which include items that tap anomie as both a state of mind and a state of society. We therefore expected to find a relationship of moderate strength between PAS and these established measures of anomie.

Furthermore, PAS should be associated with a general measure of social instability. Specifically, we expected to find a significant association between PAS and societal unease, a concern that fundamental aspects of the society such as political power, trust in community and fellow citizens, cohesion, and social and economic security are in decline [30].

To examine discriminant validity, we included a range of variables that should be distinct from PAS. Given that anomie focuses on the internal state of society, rather than threats to it from the outside, we measured collective angst—a fear that the future vitality of society is being jeopardized by outsiders [91, 92]. We predicted that PAS would, at most, be only weakly related to this type of collective angst.

We also aimed to demonstrate that the PAS, with its focus on the perceived state of society, is distinct from constructs assessing stable individual differences. Thus, we predicted that PAS would have low to moderate correlations with individual difference constructs such as social dominance orientation [93], perfectionism (i.e., having high personal standards and expectations, see [94, 95]), pessimism [96], and belief in a just world [97]. Similarly, there should only be low to moderate correlations between PAS and constructs that measure personality traits such as the Big Five (extraversion, agreeableness, conscientiousness, emotional stability, and openness to experience, [98]). Finally, we explored whether PAS might correlate with an individual’s educational level, gender, and political orientation.

### Ethics statement

This study obtained ethical clearance from the School of Psychology at the University of Queensland. The study’s procedure and aims were explained in the information sheet and participants were asked for their consent by ticking a box. Participants were told that participation was voluntary and that they were free to withdraw from the study at any time without penalty. They were debriefed at the end of the survey.

### Participants

In this study, we recruited 285 participants from the USA through Mechanical Turk. They ranged in age from 19 to 77 (*Mean* = 22.40; *SD* = 11.79; 133 female).

### Measures

The survey consisted of the same measures used in the convergent validity studies (Studies 2a-2b) including collective helplessness and hopelessness (*α* = .91), social cohesion (*α* = .84), and dangerous and threatening worldview (*α* = .86). In addition to our PAS scale (*α* = .87), we included two other anomie scales: the anomie scales developed by Srole [14] and Agnew [75]. We also included measures of societal unease [30], collective angst about the threat posed by outsiders [91, 92], social dominance orientation [99], perfectionism [95], pessimism [96], belief in a just world [100], the Big Five [98], and political orientation. For all scales participants were asked to provide their response on a 7-point scale (1 = *strongly disagree*; 7 = *strongly agree*).

*Srole’s [14] anomie scale* includes five items measuring five distinct components. Since we adapted two of the items from Srole in our anomie scale including ineffectiveness of politicians (item 10 in Table 1) and lack of trust in others (item 4 in Table 1), we did not include these two items when calculating the mean score of the Srole’s scale. Three items remained which assess individuals’ state of mind and are concerned with perceived lack of hope for the future, lack of interest to pursue future goals, and loss of internalized values and meaninglessness. An example item is “Nowadays a person has to live pretty much for today and let tomorrow take care of itself” (α = .74).

*Agnew’s [75] anomie scale* consists of eight items based on Srole’s [14] theoretical framework that anomie involves self-to-other alienation (i.e., anomie as a state of mind). This scale assesses anomie as a one-dimensional construct depicting components of normlessness, powerlessness, and despair. We excluded two items from this scale that were adapted in our own scale (items 4 and 10 in Table 1). Two examples of the remaining items are “I have had more than my fair share of worries”, and “I don’t blame anyone for trying to grab all s/he can get in this world” (α = .68).

*Societal unease* consists of 10 items that refer to the instability of society, drawing on six major factors including distrust, loss of ideology, decline in political power, decline in sense of community, socioeconomic vulnerability, and pessimism (e.g., “In our country there is not enough attention to people who are less well-off” and “American politics has a decreasing say in matters important to citizens”, [30]) (α = .62). To adapt this to a North American context, one item relating to the control of European Union over EU countries was removed.

*Collective angst about the threat posed by outsiders* was measured using Wohl and Branscombe’s [91] scale, which consists of five items (e.g., “I think the future of the American way of life is under threat from abroad”, “I am concerned about the external threats to the American way of life”). Higher scores refer to higher collective angst about outside threats (α = .95).

*Social dominance orientation* was measured using the eight-item shortened version of the SDO scale developed by Schmitt et al. ([99], e.g., “It’s OK if some groups have more of a chance in life than others”). Higher scores indicate higher levels of social dominance orientation (α = .82).

*Perfectionism* was measured using the perfectionism scale of Frost et al. [95], drawing on two dimensions of perfectionism relating to personal standards and a preference for order and structure. The scale consists of 13 items whereby ‘personal standards’ was measured with items such as “I expect higher performance in my daily tasks than most people”. ‘Preference for order and structure’ was assessed with items such as “I try to be an organized person”. The reliability of the scale was high when both subscales were combined (α = .90).

*Pessimism* was measured using the Extended Life Orientation Test (ELOT, [96]) which includes nine items capturing a tendency to expect negative outcomes (e.g., “If something can go wrong for me, it will” and “Things never work out the way I want them to”, α = .95). Higher scores indicate higher pessimism.

*Belief in a just world* was measured using a seven-item measure developed by Lipkus [100] (e.g., “I feel that people get what they deserve” and “I basically feel that the world is a fair place”, α = .94).

*Big Five personality factors* were measured using a brief 10-item measure [98]. This scale consists of five dimensions including extraversion, agreeableness, conscientiousness, emotional stability, and openness to experience.

*Demographics* included level of education and political orientation. Three items were used to measure three separate aspects of political orientation. Participants were asked to indicate their political beliefs on a seven-point Likert response format from left/liberal to right/conservative on issues of economy (e.g., social welfare, government spending, tax cuts) and social issues (e.g., immigration, homosexual marriage, abortion). The last item asked participants to indicate how close they felt toward the USA’s two major political parties in seven-point response format (1 = *democrats*; 7 = *republicans*).

### Results

As can be seen in Table 3, similar to Study 2a and 2b, there was a moderate to high relationship between PAS and constructs that should be theoretically related to anomie including collective helplessness and hopelessness (*r* = .59, *p* < .001), cohesion (*r* = -.73, *p* < .001), and dangerous and threatening worldview (*r* = .46, *p* < .001). With regard to previous anomie scales, PAS was positively and moderately related to Srole’s [14] anomie scale (*r* = .55, *p* < .001) and Agnew’s [75] anomie scale (*r* = .38, *p* < .001). Further confirming the convergent validity of PAS, there was a high positive relationship between PAS and societal unease (*r* = .71, *p* < .001).

**Table 3:** Descriptives and bivariate correlations (Study 2c).

Variables	α	Mean	SD	BSF	BL	Anomie
BSF	.80	4.06	1.02	-	-	-
BL	.86	4.65	1.11	.49***	-	-
Anomie	.87	4.36	.92	.85***	.88***	-
Helplessness and Hopelessness	.91	3.89	1.30	.55***	.47***	.59***
Cohesion	.84	4.08	1.04	-.72***	-.54***	-.73***
Dangerous World View	.86	3.88	1.24	.50***	.31***	.46***
Anomie Srole [14]	.74	4.25	1.16	.63***	.56***	.69***
Anomie Agnew [75]	.68	4.10	1.01	.58***	.35***	.54***
Societal Unease	.62	4.55	.96	.63***	.59***	.71***
Collective Angst about the threat posed by outsiders	.95	3.91	1.68	.20**	-.00	.10
Social dominance Orientation	.82	2.55	1.37	.13*	-.05	.04
Perfectionism	.90	4.62	1.02	-.06	-.13*	-.11
Pessimism	.95	3.13	1.36	.41***	.20***	.35***
Belief in just world	.94	3.88	1.32	-.27***	-.38***	-.36***
Emotional Stability	-	3.80	.77	-.12	-.11	-.13*
Extraversion	-	2.77	1.19	.31***	.16**	.26***
Conscientiousness	-	2.68	1.30	.32***	.23***	.32***
Agreeableness	-	4.48	1.76	.29***	.23***	.30***
Openness	-	4.13	1.16	.21***	.16**	.20**
Age	-	37.40	11.79	-.10	-.01	-.06
Education	-	5.53	.94	-.10	-.08	-.10
Political attitude (social issues)	-	3.61	1.82	.09	.07	.08
Political attitude (economic issues)	-	3.11	1.82	.06	-.06	-.04
Political attitude (political party)	-	3.24	1.77	.12*	.08	.09

BSF, breakdown of social fabric; BL, breakdown of leadership.

*at *p* < .05

** at *p* < .01

*** at *p* < .001.

Note. The Big-Five scale has only two items to measure each factor and this is probably the primary reason why the internal consistencies for the separate factors were rather low. It should be noted that the original Gosling *et al*.’s [98] paper warns against diminished reliability of this very short scale. Given that our primary interest is not personality assessment, we analyzed and reported the composite of the two items.

We conducted six exploratory factor analyses to examine whether PAS is different from other anomie scales and related measures. We entered the PAS items with other measures separately in a series of factor analyses (using direct oblimin rotation and principle component analysis). By and large, the results of exploratory factor analyses confirmed the two-factorial structure of anomie and demonstrated that PAS distinctively loaded on two factors separate from Srole’s anomie scale, Agnew’s anomie scale, collective helplessness and hopelessness, dangerous and threatening worldview, and cohesion. The measure of societal unease and dangerous worldview did not load on a distinct factor, but did not solely load with PAS. The results of the factor analyses confirm that PAS is distinct from other anomie measures or theoretically related constructs.

Regarding discriminant validity, the results showed that PAS was distinct from theoretically unrelated measures. There was a non-significant relationship between PAS and collective angst about the threat posed by outsiders (*r* = .10, *p* = .117). Furthermore, PAS was not correlated with social dominance orientation (*r* = .04, *p* = .560) and individual difference measure such as perfectionism (*r* = -.11, *p* = .058). The results also showed that PAS moderately and positively correlated with pessimism (*r* = .35, *p* < .001) and negatively with belief in a just world (*r* = -.36, *p* < .001). PAS had low and negative correlation with emotional stability (*r* = -.13, *p* = .027) and low to moderate positive correlations with extraversion (*r* = .26, *p* < .001), conscientiousness (*r* = .32, *p* < .001), agreeableness (*r* = .30, *p* < .001), and openness to experience (*r* = .20, *p* = .001). Finally, anomie was not related to gender (*r* = -.09, *p* = .129), age (*r* = -.06, *p* = .362), education (*r* = -.10, *p* = .097), political orientation (*r* = -.04, *p* = .578; *r* = .08, *p* = .179 in economy and social issues respectively, ranging from left to right political ideology), or voting preference (*r* = .09, *p* = .137). The result of discriminant validity suggests that anomie is not simply the product of individual differences such as the tendency to dislike imperfect situations, the belief in a just world or the tendency to experience negative emotion.

### Discussion

Study 2c provides further evidence for the convergent validity of PAS and novel evidence for its discriminant validity. We replicated the result of Studies 2a-2b, showing significant relationships between PAS and collective helplessness and hopelessness, dangerous and threatening worldview, and social cohesion. We also found that PAS was significantly related to established measures of anomie that conflate the measurement of anomie with its outcomes and anomie-related collective-level constructs such as societal unease. The results of factor analyses also confirmed that PAS is distinct from other measures and constructs.

Furthermore, we found evidence for the discriminant validity of PAS by showing that PAS was distinct from other measures of negativity such as collective angst about the threat posed by outsiders, and individual differences measures including social dominance orientation, personality traits, and political attitudes.

## The Relation between Anomie and Indicators of Societal Stability: Study 3a

Next, in Study 3a, we examined whether indicators of the social and economic stability of a society are associated with anomie as measured by PAS. Drawing on samples taken from 28 countries, we predicted that PAS would be able to differentiate countries and that there would be a positive relationship between PAS and country-level indicators tapping the malfunctioning of a social system. In particular, we focused on country-level indicators of corruption (indicating ineffective and often unfair leadership, see [101]), economic inequality [31], poverty [8], socioeconomic status [8, 74, 102], and unemployment [2, 8, 102]. Since economic instability and upheaval are predicted to fuel anomie perceptions [19], we predicted that there would be a negative relationship between PAS and economic performance indicators such as Gross Domestic Product (GDP) per capita. In addition, there should be a negative relationship between PAS and indicators that depict a healthy and functioning society, such as indices tapping the standard of living [2, 102], quality of life, and equality [8, 103].

### Ethics statement

This study obtained ethical clearance from the School of Psychology at the University of Queensland. In addition to this ethical clearance, covering data collection in all countries, further ethics approvals were also obtained from countries that have ethics committees in place: Canada (Research Ethics Board, Carleton University), Chile (School of Psychology, Pontificia Universidad Católica de Chile), China (School of Psychology and Cognitive Science, East China Normal University), Japan (School of Psychology, Nagoya University), the Netherlands (Ethical Committee for Psychology, University of Groningen), Poland (Institute of Psychology, University of Gdansk), Singapore (Institutional Review Board, Nanyang Technological University), South Africa (Human Research Ethics Committee [non-medical], University of the Witwatersrand), UK (School of Psychology, University of Exeter), and the US (Institutional Review Board for Research Involving Human Subjects, University of Tennessee). Although the remaining countries (Belgium, Brazil, Denmark, Finland, France, Germany, Hungary, India, Indonesia, Iran, Italy, Latvia, Malaysia, Pakistan, Portugal, Spain, Switzerland) do not have ethical committees, data were collected in line with standard ethical guidelines, including reassuring participants of the confidentiality of responses, their anonymity, and their right to withdraw without penalty. The study procedure and aim were explained in the information sheet and participants were informed that by continuing with the questionnaire they were indicating their consent.

### Participants

A total of 6112 undergraduate university students residing in 28 countries were recruited from North America (Canada, and the US [one data set from Tennessee and one from Northern California]), South America (Chile, Brazil), Europe (Netherlands, UK, Spain, Italy, Germany [one dataset from former East Germany and one from former West Germany], France, Denmark, Finland, Switzerland, Belgium, Portugal, Poland, Hungary, Latvia), Asia (China, Japan, Malaysia, Singapore, Indonesia, India, Pakistan), Middle East (Iran), Africa (South Africa), and Oceania (Australia). The original version of the survey was prepared in English and was translated into the native languages of the respective countries if necessary using either back-translation or panel methods. Data were collected using either online platforms or hard copy versions of the questionnaires. The data collection process started in January 2014 and ended in February 2015. The mean age of the total sample was 22.48 (*SD* = 6.40; 65% female, see Table 4).

**Table 4:** Country-level descriptive statistics (Ordered based by PAS score).

Country	N	% female	Age (Mean)	BSF (Mean)	BL (Mean)	PAS (Mean)	α of PAS	C. btw Dim	Language of Questionnaire
Pakistan	150	0	18.92	5.03	5.28	5.15	.59	.18*	Urdu
South Africa	451	81	21.04	4.69	5.04	4.87	.78	.31***	English
Poland	180	72	27.72	4.32	5.37	4.85	.83	.42***	Polish
Hungary	160	18	24.75	4.74	4.92	4.83	.87	.48***	Hungarian
Italy	156	62	25.87	4.54	5.06	4.80	.79	.28***	Italian
Brazil	146	62	23.99	4.47	5.09	4.78	.71	.27***	Portuguese
Spain	277	73	35.66	4.04	5.45	4.74	.75	.15*	Spanish
France	150	83	19.53	4.74	4.61	4.68	.76	.28***	French
Iran	170	54	22.49	4.77	4.51	4.64	.79	.37***	Persian
Latvia	149	53	23.44	4.42	4.84	4.63	.75	.26**	Latvian
Portugal	160	71	22.24	4.10	5.18	4.63	.79	.12	Portuguese
India	145	66	20.47	4.79	4.41	4.59	.65	.30***	English
Chile	151	33	20.64	4.47	4.60	4.53	.78	.21**	Spanish
Japan	382	57	18.79	3.96	4.86	4.41	.77	.32***	Japanese
US, California	141	65	23.12	4.24	4.57	4.40	.79	.34***	English
Indonesia	557	77	21.42	4.12	4.61	4.37	.72	.31***	Indonesian
Malaysia	112	85	23.20	4.43	4.29	4.35	.77	.41***	Malay
Belgium	242	22	20.37	4.24	4.44	4.34	.78	.42***	French
US, Tennessee	178	46	19.41	4.15	4.39	4.27	.80	.39***	English
China	151	79	21.62	4.21	4.06	4.14	.83	.48***	Mandarin
Germany, East	147	72	22.14	4.00	3.95	3.97	.75	.30***	German
Australia	149	72	22.17	3.59	4.28	3.94	.77	.23**	English
Germany, West	175	69	21.97	3.94	3.93	3.93	.79	.29***	German
UK	74	76	19.50	3.69	4.08	3.87	.83	.40***	English
Singapore	193	66	21.66	4.16	3.44	3.80	.82	.29***	English
Canada	233	77	20.35	3.44	4.03	3.73	.83	.33***	English
Netherlands	208	79	19.35	3.77	3.69	3.73	.79	.39***	Dutch
Finland	113	77	25.58	3.65	3.73	3.69	.81	.44***	Finnish
Denmark	164	71	22.68	3.48	3.69	3.59	.87	.55***	Danish
Switzerland	448	64	24.13	3.68	3.46	3.57	.83	.42***	French

N, number of participants; BSF, breakdown of social fabric; BL, breakdown of leadership; C. btw Dim, correlations between dimensions.

*at *p* < .05

** at *p* < .01

*** at *p* < .001.

### Measures

In addition to PAS (α = .83), we included indices of economic and social stability in each country.

### Indicators of Social Stability

*The Human Inequality Index* developed by United Nations Development Programme (UNDP) refers to the level of inequality in health, education and income [104]. It measures the estimated inequalities in the distribution of education facilities, health facilities, and income inequality. Higher coefficients indicate higher human inequality.

*The Human Development Index (HDI)* was used for the year 2013 [104] as a measure of a country’s social and economic development. The HDI is used to rank countries using a value between 0 and 1. The value is composed of life expectancy, education, and national income. A higher coefficient indicates a higher rate of human development.

### Indicators of Economic Stability

*The Inequality-adjusted HDI (IHDI)* combines two related measures of inequality and human development (the HDI as above) and accounts for the loss in HDI due to inequality. The IHDI looks beyond the average performance of a country and is considered to be a better indicator of the potential for human development as it takes into account the distribution of resources [104]. A higher coefficient indicates a higher rate of human development and a lower rate of inequality.

*Poverty* was measured using the poverty index retrieved from The World Factbook [105]. This index provides the percentage of the population below the poverty line based on indicators such as financial security and the availability of basic necessities (e.g., food, water, education, and healthcare). A higher score indicates a higher rate of poverty.

*Transparency (CPI*, *Corruption Perception Index)* shows the annual rate of corruption in a country based on expert assessments and opinion surveys [106]. A higher coefficient indicates a lower rate of corruption and a higher rate of transparency.

*The control of corruption index* is used by the World Bank to assess the strength and effectiveness of corruption prevention measures [106]. This index is calculated based on 22 different assessments and surveys [107]. A higher score indicates higher control of corruption.

*Gross Domestic Product (GDP) per capita* is the value of all domestic goods and services divided by the average (or midyear) population [107]. The values are converted at a market exchange rate to USD. The GDP per capita shows a country’s standard of living, with higher scores indicating a higher standard of living.

*Unemployment* was assessed using The World Factbook’s unemployment rate [105]. This is calculated based on the number of people who are unemployed but have the capacity for employment.

*The Youth Unemployment index* is a similar index to the unemployment coefficient from the World Factbook, but focuses on the percent of the total unemployed labour forces aged 15–24 during 2013 [105].

### Statistical Analysis

We analyzed relationships using multilevel modelling to obtain unbiased standard errors since the data have a clustered structure, with individuals being nested within countries. We assessed how PAS varies across countries and how it is associated with indicators of the health of a society across the 28 countries. To test this, we measured anomie at the individual level using PAS and then included country-level indicators of social and economic stability. In a series of separate models using Stata 12, we examined whether each of the indicators was related to anomie.

### Results

Descriptive statistics of the data and sample characteristics per country are shown in Table 4. This table shows the number of participants for each country, the language in which the survey was presented to participants, mean age of the participants, mean PAS score, as well as mean scores on the two anomie dimensions and the correlations between them. Anomie as measured by PAS was highest in Pakistan (*M* = 5.15 out of 7) and lowest in Switzerland (*M* = 3.57) and Denmark (*M* = 3.59).

Consistent with our hypotheses, analyses of bivariate relationships revealed that PAS was related to higher human inequality, lower standard of living or reduced HDI, lower inequality adjusted HDI, higher poverty, lower transparency, lower corruption control, reduced GDP per capita, and higher unemployment (see Table 5).

**Table 5:** Bivariate correlation between country-level indicators of the social and economic stability and PAS at the country-level (N = 30).

	Breakdown of Social fabric	Breakdown of leadership	PAS
Human Inequality Index	.67***	.39 (p = .052)	.56**
Human Development Index (HDI)	-.69***	-.43*	-.60***
Inequality Adjusted HDI	-.70***	-.40*	-.59**
Poverty	.44*	.50**	.53**
Transparency (Corruption Index)	-.76***	-.61***	-.76***
Corruption control	-.77***	-.59**	-.74***
GDP per capita	-.74***	-.63***	-.75***
Unemployment	.28	.58**	.51**
Youth unemployment	.25	.63***	.53**

* *p* < .05 (two-tailed)

** *p* < .01 (two-tailed)

*** at *p* < .001.

We next conducted a multilevel regression analysis (see Table 6), entering country-level variables as predictors in 10 separate models to examine the relationship between country-level indicators of the social and economic stability (as independent variables) and individuals’ PAS score (as the dependent variable). These country-level indicators were entered in separate models because some of the indicators of social and economic stability were highly correlated, making it problematic to include them together in a single model (this is because with 12 correlations higher than .75 [between .78 to .96] among 9 indicators of social and economic instability, problems with multicollinearity would otherwise arise). We found that country-level indicators of social and economic stability including HDI, inequality adjusted HDI, transparency (corruption index), corruption control, and GDP per capita negatively predicted PAS. On the other hand, human inequality, poverty, unemployment in the general population, and youth unemployment positively predicted PAS, implying that higher poverty, unemployment, and inequality was associated with higher anomie.

**Table 6:** Multilevel regressions predicting PAS.

Parameters	Intercept	Coefficient
Model 1: Country level predictors	4.33***	-
Model 2: Human Inequality Index	3.91***	.03**
Model 3: HDI	6.49***	-2.60***
Model 4: Inequality Adjusted HDI	5.70***	-1.89***
Model 5: Poverty	3.86***	.03**
Model 6: Transparency (Corruption index)	5.59***	-.02***
Model 7: Corruption control	4.64***	-.33***
Model 8: GDP per capita	4.81***	-.01***
Model 9: Unemployment	3.98***	.04**
Model 10: youth unemployment	3.99***	.02**

** at *p* < .01

*** at *p* < .001.

### Discussion

Drawing on a large cross-cultural sample we found that PAS meaningfully differentiated countries in terms of anomie in expected ways. We found that perceptions of anomie were lower in countries that are known to be socially stable (e.g., Switzerland, Denmark, Finland, the Netherlands, Canada), and higher in countries that were hard hit by recent economic crises (e.g., Portugal), countries with fast-growing economies and thus undergoing rapid social change (e.g., Brazil, India), countries that face internal conflict and unrest (Pakistan), and countries that have experienced massive structural changes during recent decades (e.g., Iran, South Africa).

These results provide some initial evidence that PAS is able to differentiate countries in meaningful ways. Importantly too, intuitive expectations about which countries should score high and low in anomie were further confirmed in analyses examining the relationship between anomie and indicators of economic and social stability. In Study 3a, we found that the indicators of corruption, standards of living, economic and human inequality, and economic condition (e.g., poverty and unemployment) were predictably related to anomie as measured by PAS. In sum, and lending strong support to the validity of PAS, perceptions of anomie varied across different social contexts and this variance can be explained in meaningful ways by societal functioning indicators.

## Predictive Validity: Study 3b

As outlined before, anomie has been found to be associated with reduced well-being and lower life satisfaction [58, 61, 64]. As such, we predicted that there would be a negative relationship between anomie as measured by PAS and life satisfaction. Furthermore, because individuals living in societies characterised by high anomie will tend to withdraw from the superordinate group, anomie should be associated with reduced identification with one’s country [1, 74]. Evidence for these relationships would provide greater confidence in the predictive validity of PAS.

### Measures

In the same cross-cultural sample reported in Study 3a, we measured national identification and life satisfaction. Responses were recorded on seven-point Likert scales with endpoints ranging from 1 (*strongly disagree)* to 7 (s*trongly agree)*.

*National Identification* was assessed with four items adapted from Jetten, Spears, and Manstead [108]. An example item was “I identify with my country” (α = .86).

*Life Satisfaction* was measured using a five-item satisfaction with life scale [109]. Example items were “I am satisfied with my life” and “In most ways my life is close to my ideal” (α = .83).

### Results

**Fig 1:**
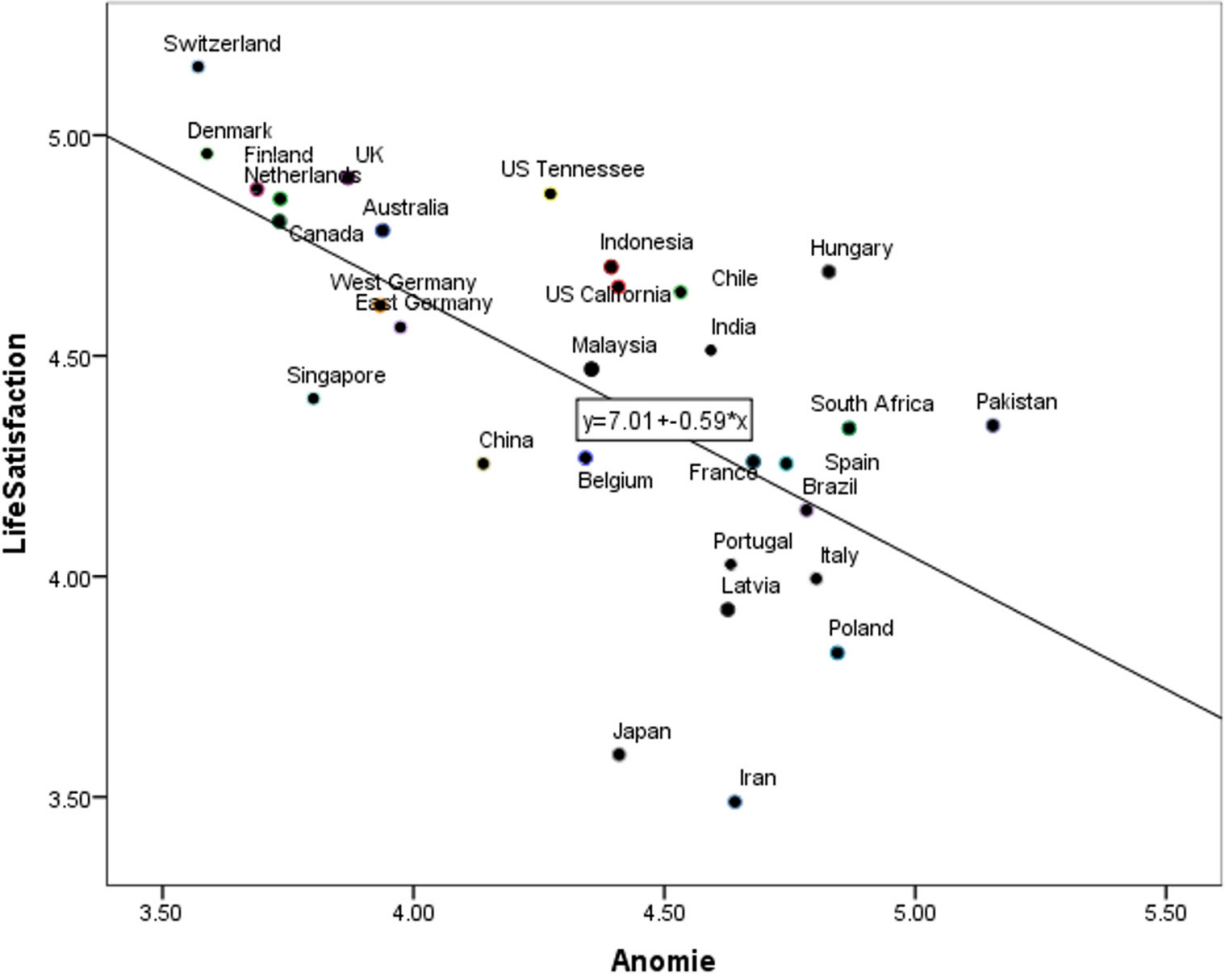
Relation between PAS and life satisfaction across countries.

**Fig 2:**
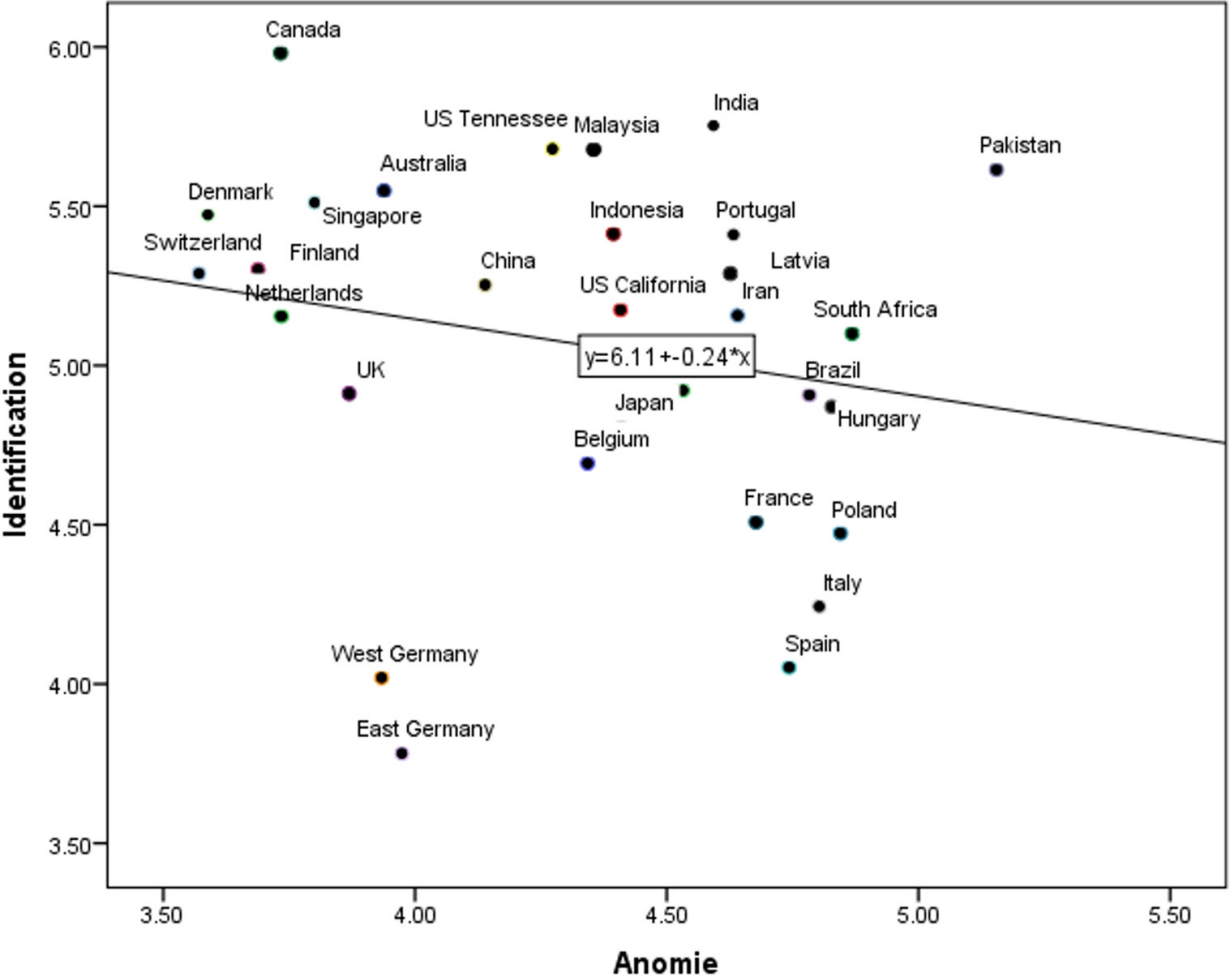
Relation between PAS and identification across countries.

Pearson correlations revealed that higher PAS was related to lower life satisfaction (*r* = -.28, *p* < .001, R^2^ Linear = -.59, see Fig 1) and lower national identification (*r* = -.29, *p* < .001, R^2^ Linear = -.24, Fig 2).

We next examined the relationship between PAS and the predicted outcomes by accounting for the variance of PAS between countries using multilevel modelling. To do so, we first centered individuals’ PAS scores with regards to their respective countries’ mean PAS score. This is crucial for the interpretation of the intercept and slope parameters in a multilevel analysis [110]. Next, using unstructured maximum likelihood estimation variance we entered the PAS score at the individual level (fixed-effect parameters) as a predictor and centered PAS at the country level (random-effect parameters) as a between-country variation factor. By introducing centered PAS as a random component, we are effectively allowing the slope to be different for each country, estimating a separate regression line within each country.

We conducted two separate models to investigate the effect of PAS on the predicted outcomes. In the first model we included life satisfaction as the level 1 dependent variable, PAS as the level 1 independent variable, country as the level 2 variable, and centered PAS as a random-effects parameter at level 2. This revealed a significant model (*χ*^*2*^ (1) = 169.11, *p* < .001) with PAS (level 1 PAS) significantly predicting life satisfaction (*B* = -.31, *p* < .001). The analyses yielded an intra-class correlation coefficient (ICC) of .34 for life satisfaction. In other words, 34 percent of individuals’ life satisfaction was explained by centered PAS at the country level. In the second model we examined the relationship between PAS and national identification. The model was significant, *χ*^*2*^ (1) = 134.37, *p* < .001, and PAS significantly predicted national identification, *B* = -.46, *p* < .001. The analyses yielded an intra-class correlation coefficient (ICC) of .45.

We further examined whether PAS explained more variance in these predicted outcomes compared to the country-level indicators of social and economic stability in Study 3a. To achieve this, first we entered life satisfaction as the dependent variable and PAS along with other social and economic stability indicators as independent variables at level 1 in nine separate models (see Table 7). We used these nine models in order to avoid multicollinearity problems associated with the indicators of social and economic stability (12 correlations higher than .78 exist among the nine indicators of social and economic instability). We included country and PAS centered score at level 2. The result revealed that out of all independent variables at level 1 across nine models, PAS was the only and the strongest predictor of life satisfaction (*B* = -.30 to -.33, *p* < .001, see Table 7). Next, we repeated the same analysis for national identification. The result showed that PAS was most strongly associated with national identification (*B* = -.44 to -.47, *p* < .001); while four country-level indicators of the social and economic stability were also significantly related to national identification, there were so to a lesser extent (human inequality: *B* = .04, *p* = .004; HDI: *B* = -2.83, *p* = .001, inequality adjusted HDI: *B* = -2.20, *p* = .001, corruption control: *B* = -.20, *p* = .026, see Table 7).

**Table 7:** Multilevel regression predicting life satisfaction and national identification.

		Model A	Model B
Parameters		DV: Life satisfaction	DV: identification
Model 1a, 1b	PAS	-.33***	-.46***
	Human inequality	-.01	.04**
Model 2a, 2b	PAS	-.32***	-.47***
	HDI	.24	-2.83**
Model 3a, 3b	PAS	-.33***	-.47***
	Inequality adjusted HDI	.47	-2.20**
Model 4a, 4b	PAS	-.30***	-.44***
	Poverty	-.01	.01
Model 5a, 5b	PAS	-.31**	-.46***
	Corruption	.01	-.01
Model 6a, 6b	PAS	-.31***	-.47***
	Corruption control	.09	-.20*
Model 7a, 7b	PAS	-.31***	-.46***
	GDP per capita	4.89	-7.90
Model 8a, 8b	PAS	-.32***	-.45***
	Unemployment	-.01	-.01
Model 9a, 9b	PAS	-.32***	-.45***
	Youth unemployment	-.01	-.01

* at *p* < .05

** at *p* < .01

*** at *p* < .001.

### Discussion

Study 3b focused on the predictive validity of PAS. We examined the effect of PAS on life satisfaction and national identification. At the individual level we found that higher PAS was associated with lower life satisfaction and lower national identification. Additionally, at the country level we found that aggregate perceptions of anomie were associated with both lower well-being and lower identification with the superordinate group. The latter results provide good evidence that it is anomie as a state of society and not just as a state of mind that is related to psychological outcomes. PAS also predicted these outcomes over and above the country-level social and economic indicators, highlighting the validity of focusing on *perceived* state of society rather than on social and economic indicators of society.

## General Discussion

We set ourselves the task to develop a valid measure of anomie that would improve on previous scales by measuring anomie without conflating it with its individual-level outcomes. Relying on classical sociological theorizing on anomie [9–11, 13], we consider anomie as a perceived state of society (also see [42]) and developed the Perception of Anomie Scale (PAS). This scale aims to capture the two basic dimensions of anomie identified by Durkheim [13]: the perception of breakdown of social fabric and the perception of breakdown of leadership.

We conducted six studies examining the psychometric properties of PAS. We first confirmed PAS’s factor structure using exploratory and confirmatory factor analysis (Studies 1a-1b). We then provided evidence for the scale’s convergent and discriminant validity (Studies 2a-2c). As evidence for convergent validity, we showed in Studies 2a-2c that PAS correlated moderately to highly with theoretically related measures. Specifically, PAS was positively correlated with other measures that also capture emotions and beliefs related to the state of society such as collective hopelessness, collective helplessness, the endorsement of a worldview that society is a dangerous and threatening place, and low perceived social cohesion (the latter was measured only in Study 2a and 2c). Providing further evidence for the convergent validity of PAS, in Study 2c it was found that PAS was also significantly and positively correlated with previous measures of anomie and societal unease. Evidence for the discriminant validity of PAS was obtained in Study 2c. Here we showed that PAS is distinct from other measures from which it should theoretically be distinct, such as collective angst about the threat posed by outsiders, individual differences in social dominance orientation, personality traits, and demographics including political attitudes, gender, age, and education.

In the final two studies, we found that PAS maps onto macro-level indicators of social and economic stability and that it predicts individual-level outcomes. In study 3a, we found that PAS corresponds with indicators of the social and economic stability of a country including corruption, standard of living, economic and human inequality, poverty, and unemployment. PAS differentiated countries in a meaningful way: PAS scores were lower in countries that are commonly described as stable (e.g., Denmark, Switzerland, see [111]; or [107] for Worldwide Governance Indicators) in comparison with countries that are undergoing massive structural changes (e.g., Iran, South Africa) or countries that have been hard hit by the recent global financial crisis (e.g., Spain, Portugal). In Study 3b, we aimed to obtain evidence for the predictive validity of PAS. Across 28 countries, PAS significantly predicted theoretically relevant outcomes including life satisfaction and dis-identification from the superordinate group (i.e., national identification). Using multilevel modelling, we found that PAS makes a unique contribution in explaining variance in these individual-level outcomes over and above country-level indicators of social and economic stability. This finding lends support to the importance of focusing on perceptions of society and their role in predicting the individual-level outcomes of anomie.

### Implications

By developing a new measure of anomie that separates the anomie construct from its consequences, we provide a more valid measure of anomie than has previously been available, gaining a number of important opportunities for advancing the current state of social research.

First, as a measure of societal instability, PAS can provide the opportunity to gain a better understanding of the context of failing or troubled societies. This allows for theory development that is not possible when focusing only on stable social systems. Specifically, theoretical frameworks that focus on perceptions of the state of society, such as social identity theory [112], system justification theory [113, 114], and social dominance theory [93], have been developed to explain group processes and intergroup relations against the backdrop of stability and consensus on the status of various groups in society. In these theories, even if low-status groups challenge the status quo and engage in collective protest to change status relations, they do so in recognition that there is a status quo to be challenged and often within a largely orderly and functioning society. However, in social contexts characterised by high anomie, political or economic crises, war, revolutions, rapid social change, or high instability and chaos destabilize any previously existing status quo [2, 8].As a result, the aforementioned theoretical frameworks are ideally suited to explain processes that are evident in the context of relative stability but fall short of explaining processes typically observed in social contexts with high levels of anomie. With PAS, we are in a better position to assess the features of these contexts that contribute to anomie, facilitating new insights into previously understudied societal processes.

Second, the development of PAS provides novel insights into human behavior in destabilized and atomized social contexts. Drawing on the broader body of knowledge in our conceptual framework, we are in a good position to develop an analysis of anomie that accounts for how individual- and societal-levels of analysis relate to each other and possibly interact [42]. More precisely, by measuring the *perception* of the *state of society*, PAS differentiates the understanding of the how people think about their societies from the individual outcomes that arise in response to perceived anomie. This not only solves the longstanding problems with operationalizing the concept of anomie [19] but also serves as an impetus for future research on how, why, and when anomie arises, and how it can be prevented or repaired.

### Future Research and Limitations

With a better tool to capture the anomie construct *per se*, researchers can address a number of novel and important research questions. First, by decoupling the anomie construct from its consequences, we can study their relationship more accurately and precisely. This is important as there are large seas of uncharted territory in our understanding of anomie. For instance, although the negative well-being consequences of anomie have been documented, processes relating to the perception of anomie are poorly understood. In the present study we demonstrated that anomie is associated with lower identification with the superordinate group (see Study 3b). In line with previous research, we predict that when the superordinate group is viewed as a source of negative identity incapable of achieving social harmony across different subgroups, these larger social psychological structures are likely to schism and break apart [115], escalating intergroup tensions and chaos. This work would also have the potential to provide insights into the kinds of social factors that may motivate the development of extremist groups.

Future research should also examine other consequences of anomie at the societal level. For example, the relationship between anomie and the rise of authoritarianism [65, 116], and the rising popularity of autocratic groups and parties when more democratic processes or groups are perceived to be failing (e.g., rise of Nazism, see [116, 117]) are well-established in the literature. PAS provides a valid and reliable tool to further analyse this relationship and specifically focusing on the process by which the transition between democracy and tyranny takes place.

Our research mainly focused on validating a measure of anomie, but future studies should be wider in scope and, using PAS, examine how anomie develops and evolves within a society. For instance, in our theoretical framework we suggest that anomie involves the interaction between the breakdown of social fabric and the breakdown of leadership (also see [42]). Although the interaction between the two dimensions was not examined in the current research, interaction may play a significant role in the development of anomie. For instance, perceived lack of trust and consensual moral standards within a given society could undermine the collective consensus that the leadership is legitimate and effective. On the other hand, the perception of illegitimate and ineffective leadership may undermine the perception that people can be trusted. It would be useful to investigate whether and how such interactive processes can trigger an increase in anomie, an understanding of which may be important in considering ways to prevent or address anomie.

### Conclusion

The main aim of this research was to develop and validate a new scale to measure anomie. Having achieved this, we are now better equipped to study contexts where societies are crumbling and unstable. Just as importantly, bringing this classic concept under closer scrutiny and developing a scale to measure it is an important first step for further theory building on how the state of society affects individuals (for a similar argument, see [36]).

## References

[1] Ådnanes M. Social transitions and anomie among post-communist Bulgarian youth. Young. 2007;15(1):49–69.

[2] Genov N. Transformation and anomie: Problems of quality of life in Bulgaria. Social Indicators Research. 1998;43(1–2):197–209.

[3] Heydari A, Teymoori A, Haghish E, Mohamadi B. Influential factors on ethnocentrism: The effect of socioeconomic status, anomie, and authoritarianism. Social Science Information. 2014;53(2):240–54.

[4] Heydari A, Teymoori A, Nasiri H. Development of suicidality within socioeconomic context: Mediation effect of parental control and anomie. Omega: Journal of Death and Dying. 2014;68(1):63–76. 10.2190/om.68.1.d 24547665

[5] Lopes CA, Frade C. The way into bankruptcy: Market anomie and sacrifice among Portuguese consumers. Journal of Consumer Policy. 2012;35(4):477–96.

[6] Jamieson R. Towards a criminology of war in Europe In: Ruggiero V., South N, Taylor I, editors. The new European criminology: Crime and social order in Europe. New York, NY: Routledge; 1998 p. 480–506.

[7] Mestrovic SG, Lorenzo R. Durkheim's concept of anomie and the abuse at Abu Ghraib. Journal of Classical Sociology. 2008;8(2):179–207.

[8] Huschka D, Mau S. Social anomie and racial segregation in South Africa. Social Indicators Research. 2006;76(3):467–98.

[9] Merton R. Social structure and anomie. American Sociological Review. 1938;3(5):672–82.

[10] Merton R. Social theory and social structure New York, NY: Free Press; 1968.

[11] Messner SF, Rosenfeld R. Crime and the American dream (3th ed). Belmont, CA: Wadsworth; 2001.

[12] Zhao LS. Anomie theory and crime in a transitional China (1978-). International Criminal Justice Review. 2008;18(2):137–57.

[13] Durkheim E. Suicide. London, UK: Routledge & Kegan Paul Ltd; 1897/1987.

[14] Srole L. Social integration and certain corollaries: An exploratory study. American Sociological Review. 1956;21(6):709–16.

[15] Davol SH, Reimanis G. The role of anomie as a psychological concept. Journal of Individual Psychology. 1959;15(2):215–25.

[16] McClosky H, Schaar JH. Psychological dimensions of anomy. American Sociological Review. 1965:14–40. 14247325

[17] Konty M. Microanomie: The cognitive foundations of the relationship between anomie and deviance. Criminology. 2005;43(1):107–32.

[18] Baumer EP. Untangling research puzzles in Merton's multilevel anomie theory. Theoretical Criminology. 2007;11(1):63–93.

[19] Bjarnason T. Anomie among European adolescents: Conceptual and empirical clarification of a multilevel sociological concept. Sociological Forum. 2009;24(1):135–61.

[20] Fischer CS. On urban alienations and anomie: Powerlessness and social isolation. American Sociological Review. 1973;38(3):311–26. 4711439

[21] Martin R. Anomie, spirituality, and crime. Journal of Contemporary Criminal Justice. 2000;16(1):75–98.

[22] Thorlindsson T, Bernburg JG. Durkheim’s theory of social order and deviance: A multi-level test. European Sociological Review. 2004;20(4):271–85.

[23] Form WH. The social construction of anomie: A four-nation study of industrial workers. American Journal of Sociology. 1975;80(5):1165–91.

[24] Bjarnason T. Parents, religion and perceived social coherence: A Durkheimian framework of adolescent anomie. Journal for the Scientific Study of Religion. 1998;37(4):742–54.

[25] Hilbert RA. Anomie and the moral regulation of reality: The Durkheimian tradition in modern relief. Sociological Theory. 1986;4(1):1–19.

[26] Travis R. The MOS alienation scale: An alternative to Srole's anomia scale. Social Indicators Research. 1993;28(1):71–91.

[27] Teevan JJ Jr. On measuring anomia: Suggested modification of the Srole scale. Pacific Sociological Review. 1975;18(2):159–70.

[28] Moore AB. An instrument to measure anomia. Adult Education Quarterly. 1980;30(2):82–91.

[29] Meštrović SG. Durkheim's concept of anomie considered as a 'total' social fact. British Journal of Sociology. 1987;38(4):567–83.

[30] Steenvoorden EH. A general discontent disentangled: A conceptual and empirical framework for societal unease. Social Indicators Research. 2015;124(1):85–110.

[31] Savolainen J. Inequality, welfare state, and homicide: Further support for the institutional anomie theory. Criminology. 2000;38(4):1021–42.

[32] Muftić LR. Advancing institutional anomie theory: A microlevel examination connecting culture, institutions, and deviance. International Journal of Offender Therapy and Comparative Criminology. 2006;50(6):630–53. 17068189 10.1177/0306624X06287284

[33] Tajfel H. Social identity and intergroup relations Cambridge, UK: Cambridge University Press; 1982.

[34] Turner JH. The state of theorizing in sociological social psychology: A grand theorist’s view In: Burke PJ, editor. Contemporary social psychological theories. Standford, California: Standford University Press; 2006 p. 353–73.

[35] David O, Bar-Tal D. A sociopsychological conception of collective identity: The case of national identity as an example. Personality and Social Psychology Review. 2009;13(4):354–79. 10.1177/1088868309344412 19700736

[36] Oishi S, Kesebir S, Snyder BH. Sociology: A lost connection in social psychology. Personality and Social Psychology Review. 2009;13(4):334–53. 10.1177/1088868309347835 19815492

[37] Chiu C-Y, Gelfand MJ, Yamagishi T, Shteynberg G, Wan C. Intersubjective culture: The role of intersubjective perceptions in cross-cultural research. Perspectives on Psychological Science. 2010;5(4):482–93. 10.1177/1745691610375562 26162194

[38] Hardin CD, Higgins ET. Shared reality: How social verification makes the subjective objective In: Sorrentino RM, Higgins ET, editors. Handbook of motivation and cognition: The interpersonal context. 3 New York, NY: The Guilford Press; 1996 p. 28–84.

[39] Moscovici S, Duveen G. Social representations: Explorations in social psychology Cambridge, UK: Polity Press; 2000.

[40] Thompson K. Moral panics London, UK: Routledge; 1998.

[41] Alexander JC. Toward a theory of cultural trauma In: Alexander J. C., Eyerman R., Giesen B., Smelser N. J., Sztompka P, editors. Cultural trauma and collective identity Berkeley, CA: University of California Press; 2004 p. 1–30.

[42] Teymoori A, Bastian B, Jetten J. Towards a psychological analysis of anomie. Manuscript under review. 2016.

[43] Dirks KT. The effects of interpersonal trust on work group performance. Journal of Applied Psychology. 1999;84(3):445–55. 10.1037/0021-9010.84.3.445 10380424

[44] Putnam RD. Bowling alone: The collapse and revival of American community New York, NY: Simon & Schuster; 2000.

[45] Uslaner EM. The moral foundations of trust Cambridge, England: Cambridge University Press; 2002.

[46] Bass BM. From transactional to transformational leadership: Learning to share the vision. Organizational Dynamics. 1990;18(3):19–31.

[47] Haslam SA, Reicher SD, Platow MJ. The new psychology of leadership: Identity, influence and power New York, NY: Psychology Press; 2011.

[48] Tyler TR. Social justice: Outcome and procedure. International Journal of Psychology. 2000;35(2):117–25.

[49] Tyler TR. A psychological perspective on the legitimacy of institutions and authorities In: John TJ, Major B, editors. The psychology of legitimacy: Emerging perspectives on ideology, justice, and intergroup relations. Cambridge, UK: Cambridge University Press; 2001 p. 416–36.

[50] Tyler TR. Psychological perspectives on legitimacy and legitimation. Annual Review of Psychology. 2006;57:375–400. 16318600 10.1146/annurev.psych.57.102904.190038

[51] Reicher S, Haslam SA, Hopkins N. Social identity and the dynamics of leadership: Leaders and followers as collaborative agents in the transformation of social reality. The Leadership Quarterly. 2005;16(4):547–68.

[52] Ambrose ML, Arnaud A. Are procedural justice and distributive justice conceptually distinct? In: Greenberg J, Colquitt JA, editors. Handbook of organizational justice. Mahwah, NJ: Lawrence Erlbaum Associates Publishers; 2005 p. 59–84.

[53] Tyler TR, Blader SL. The group engagement model: Procedural justice, social identity, and cooperative behavior. Personality and Social Psychology Review. 2003;7(4):349–61. 14633471 10.1207/S15327957PSPR0704_07

[54] Haslam SA, Reicher S. Identity entrepreneurship and the consequences of identity failure: The dynamics of leadership in the BBC Prison Study. Social Psychology Quarterly. 2007;70(2):125–47.

[55] Rothstein B, Eek D. Political corruption and social trust an experimental approach. Rationality and Society. 2009;21(1):81–112.

[56] Rothstein B. Trust, social dilemmas and collective memories. Journal of Theoretical Politics. 2000;12(4):477–501.

[57] Reicher S, Haslam SA. Rethinking the psychology of tyranny: The BBC prison study. The British Journal of Social Psychology. 2006;45:1–40. 16573869 10.1348/014466605X48998

[58] Blanco A, Díaz D. Social order and mental health: A social wellbeing approach. Psychology in Spain. 2007;(11):61–71.

[59] Lachman ME, Weaver SL. The sense of control as a moderator of social class differences in health and well-being. Journal of Personality and Social Psychology. 1998;74(3):763–73. 9523418 10.1037//0022-3514.74.3.763

[60] van der Doef M, Maes S. The job demand-control (-support) model and psychological well-being: A review of 20 years of empirical research. Work & stress. 1999;13(2):87–114.

[61] Brockmann H, Delhey J, Welzel C, Yuan H. The China puzzle: Falling happiness in a rising economy. Journal of Happiness Studies. 2009;10(4):387–405.

[62] Fritsche I, Jonas E, Kessler T. Collective reactions to threat: Implications for intergroup conflict and for solving societal crises. Social Issues and Policy Review. 2011;5(1):101–36.

[63] Leger RG. Where have all the flowers gone? A sociological analysis of the origins and content of youth values of the seventies. Adolescence. 1980;15(58):283–300. 7395588

[64] Elgar FJ, Davis CG, Wohl MJ, Trites SJ, Zelenski JM, Martin MS. Social capital, health and life satisfaction in 50 countries. Health & Place. 2011;17(5):1044–53. 21784694 10.1016/j.healthplace.2011.06.010

[65] Blank T. Determinants of national identity in East and West Germany: An empirical comparison of theories on the significance of authoritarianism, anomie, and general self-esteem. Political Psychology. 2003;24(2):259–88.

[66] Scheepers P, Felling A, Peters J. Anomie, authoritarianism and ethnocentrism: Update of a classic theme and an empirical test. Politics & the Individual. 1992;2:43–59.

[67] Axelrod R. The evolution of cooperation New York, NY: Basic Books Publishers; 2006.

[68] Das TK, Teng B-S. Between trust and control: Developing confidence in partner cooperation in alliances. The Academy of Management Review. 1998;23(3):491–512.

[69] Granovetter M. The strength of weak ties. American Journal of Sociology. 1973;78(6):1360–80.

[70] Granovetter M. The strength of weak ties: A network theory revisited. Sociological Theory. 1983;1(1):201–33.

[71] Levin DZ, Cross R. The strength of weak ties you can trust: The mediating role of trust in effective knowledge transfer. Management Science. 2004;50(11):1477–90.

[72] Sani F. When subgroups secede: Extending and refining the social psychological model of schism in groups. Personality and Social Psychology Bulletin. 2005;31(8):1074–86. 16000268 10.1177/0146167204274092

[73] Hornsey MJ, Hogg MA. Assimilation and diversity: An integrative model of subgroup relations. Personality and Social Psychology Review. 2000;4(2):143–56.

[74] van Snippenburg LB, Scheepers P. Social class and political behavior during a period of economic stagnation: Apathy and radicalism in the Netherlands, 1985. Political Psychology. 1991;12:41–63.

[75] Agnew RS. Success and anomie: A study of the effect of goals on anomie. The Sociological Quarterly. 1980;21(1):53–64.

[76] Kapsis RE. Black ghetto diversity and anomie: A sociopolitical view. American Journal of Sociology. 1978;83:1132–53. 10.1086/226677 655337

[77] Mizruchi EH. Social structure and anomia in a small city. American Sociological Review. 1960;25(5):645–54.

[78] Rushing WA. Class, Culture, and "Social Structure and Anomie". American Journal of Sociology. 1971;76(5):857–72.

[79] Winslow RW. Status management in the adolescent social system: A reformulation of Merton's anomie theory. The British Journal of Sociology. 1968;19(2):143–59. 5659806

[80] Buhrmester M, Kwang T, Gosling SD. Amazon's Mechanical Turk: A new source of inexpensive, yet high-quality, data? Perspectives on Psychological Science. 2011;6(1):3–5. 10.1177/1745691610393980 26162106

[81] Tabachnick BG, Fidell LS. Using multivariate statistics Boston: Pearson Education; 2007.

[82] Pallant J. SPSS survival manual: A step by step guide to data analysis using SPSS NSW, Australia: Allen & Unwin; 2011.

[83] De Winter JC, Dodou D. Factor recovery by principal axis factoring and maximum likelihood factor analysis as a function of factor pattern and sample size. Journal of Applied Statistics. 2012;39(4):695–710.

[84] Passas N. Continuities in the anomie tradition In: Adler FA, Laufer WS, Merton RK, editors. The legacy of anomie theory. New Brunswick, NJ: Transaction Publishers; 1995 p. 91–112.

[85] Orru M. The ethics of anomie: Jean Marie Guyau and Emile Durkheim. British Journal of Sociology. 1983;34(4):499–518.

[86] Zhao R, Cao L. Social change and anomie: A cross-national study. Social Forces. 2010;88(3):1209–29.

[87] Mazerolle L, Wickes R, McBroom J. Community variations in violence: The role of social ties and collective efficacy in comparative context. Journal of Research in Crime and Delinquency. 2010;47(1):3–30.

[88] Sampson RJ, Raudenbush SW, Earls F. Neighborhoods and violent crime: A multilevel study of collective efficacy. Science. 1997;277(5328):918–24. 9252316 10.1126/science.277.5328.918

[89] Sibley CG, Duckitt J. Big-five personality, social worldviews, and ideological attitudes: Further tests of a dual process cognitive-motivational model. The Journal of Social Psychology. 2009;149(5):545–61. 10.1080/00224540903232308 20014520

[90] Duckitt J, Wagner C, du Plessis I, Birum I. The psychological bases of ideology and prejudice: Testing a dual process model. Journal of Personality and Social Psychology. 2002;83(1):75–93. 12088134

[91] Wohl MJA, Branscombe NR. Group threat, collective angst, and ingroup forgiveness for the war in Iraq. Political Psychology. 2009;30(2):193–217.

[92] Wohl MJA, Branscombe NR, Reysen S. Perceiving your group's future to be in jeopardy: Extinction threat induces collective angst and the desire to strengthen the ingroup. Personality and Social Psychology Bulletin. 2010;36(7):898–910. 10.1177/0146167210372505 20519571

[93] Sidanius J, Pratto F. Social dominance: An intergroup theory of social hierarchy and oppression Cambridge, UK: Cambridge University Press; 2001.

[94] Stoeber J, Otto K. Positive conceptions of perfectionism: Approaches, evidence, challenges. Personality and Social Psychology Review. 2006;10(4):295–319. 17201590 10.1207/s15327957pspr1004_2

[95] Frost RO, Marten P, Lahart C, Rosenblate R. The dimensions of perfectionism. Cognitive Therapy and Research. 1990;14(5):449–68.

[96] Chang EC, Maydeu-Olivares A, D'Zurilla TJ. Optimism and pessimism as partially independent constructs: Relationship to positive and negative affectivity and psychological well-being. Personality and Individual Differences. 1997;23(3):433–40.

[97] Furnham A. Belief in a just world: Research progress over the past decade. Personality and Individual Differences. 2003;34(5):795–817.

[98] Gosling SD, Rentfrow PJ, Swann WB. A very brief measure of the Big-Five personality domains. Journal of Research in personality. 2003;37(6):504–28.

[99] Schmitt MT, Branscombe NR, Kappen DM. Attitudes toward group‐based inequality: Social dominance or social identity? British Journal of Social Psychology. 2003;42(2):161–86. 12869240 10.1348/014466603322127166

[100] Lipkus I. The construction and preliminary validation of a global belief in a just world scale and the exploratory analysis of the multidimensional belief in a just world scale. Personality and Individual Differences. 1991;12(11):1171–8.

[101] Passas N. Global anomie, dysnomie, and economic crime: Hidden consequences of neoliberalism and globalization in Russia and around the world. Social Justice. 2000;27(2):16–44.

[102] Glatzer W, Bös M. Subjective attendants of unification and transformation in Germany. Social Indicators Research. 1998;43(1–2):171–96.

[103] Messner SF. Income inequality and murder rates: Some cross-national findings. Comparative Social Research. 1980;3:185–98.

[104] Human Development Report 2014– Sustaining human progress: Reducing vulnerabilities and building resilience. United Nations Development Programme. 2014. Available: http://hdr.undp.org/en/2014-report.

[105] The World Factbook. 2013. Available: https://www.cia.gov/library/publications/the-world-factbook/.

[106] Corruption Perceptions Index. 2013. Available: http://www.transparency.org/.

[107] World Bank. Worldwide governance indicators. 2013.

[108] Jetten J, Spears R, Manstead AS. Intergroup norms and intergroup discrimination: Distinctive self-categorization and social identity effects. Journal of Personality and Social Psychology. 1996;71(6):1222–33. 8979388 10.1037//0022-3514.71.6.1222

[109] Diener E, Emmons RA, Larsen RJ, Griffin S. The satisfaction with life scale. Journal of Personality Assessment. 1985;49(1):71–5. 16367493 10.1207/s15327752jpa4901_13

[110] Enders CK, Tofighi D. Centering predictor variables in cross-sectional multilevel models: A new look at an old issue. Psychological Methods. 2007;12(2):121–38. 17563168 10.1037/1082-989X.12.2.121

[111] Fragility in the world 2014. 2014. Available: http://fsi.fundforpeace.org/rankings-2014.

[112] Tajfel H, Turner JC, editors. An integrative theory of intergroup conflict Chicago: Nelson-Hall; 1979.

[113] Jost JT, Banaji MR. The role of stereotyping in system‐justification and the production of false consciousness. British Journal of Social Psychology. 1994;33(1):1–27.

[114] Jost JT, Banaji MR, Nosek BA. A decade of system justification theory: Accumulated evidence of conscious and unconscious bolstering of the status quo. Political Psychology. 2004;25(6):881–919.

[115] Sani F. Self continuity: Individual and collective perspectives New York, NY: Taylor & Francis; 2008.

[116] Oesterreich D. Flight into security: A new approach and measure of the authoritarian personality. Political Psychology. 2005;26(2):275–98.

[117] Arendt H. The origins of totalitarianism New York, NY: Harcourt Brace Inc; 1951.

